# Proteomic analysis of Rac1 signaling regulation by guanine nucleotide exchange factors

**DOI:** 10.1080/15384101.2016.1183852

**Published:** 2016-05-06

**Authors:** Hadir Marei, Alejandro Carpy, Boris Macek, Angeliki Malliri

**Affiliations:** a Cell Signaling Group, Cancer Research UK Manchester Institute, The University of Manchester, Manchester, UK; b Proteome Center Tuebingen, Interfaculty Institute for Cell Biology, University of Tuebingen, Tuebingen, Germany

**Keywords:** Guanine nucleotide Exchange Factors (GEFs), Ingenuity Integrated Pathway Analysis (IPA), IQGAP1, P-Rex1, Rac1, Small GTPases, Stable Isotope Labeling by Amino acids in Cell culture (SILAC), Tandem Affinity Purification (TAP), Tiam1, TMOD3

## Abstract

The small GTPase Rac1 is implicated in various cellular processes that are essential for normal cell function. Deregulation of Rac1 signaling has also been linked to a number of diseases, including cancer. The diversity of Rac1 functioning in cells is mainly attributed to its ability to bind to a multitude of downstream effectors following activation by Guanine nucleotide Exchange Factors (GEFs). Despite the identification of a large number of Rac1 binding partners, factors influencing downstream specificity are poorly defined, thus hindering the detailed understanding of both Rac1's normal and pathological functions. In a recent study, we demonstrated a role for 2 Rac-specific GEFs, Tiam1 and P-Rex1, in mediating Rac1 anti- versus pro-migratory effects, respectively. Importantly, via conducting a quantitative proteomic screen, we identified distinct changes in the Rac1 interactome following activation by either GEF, indicating that these opposing effects are mediated through GEF modulation of the Rac1 interactome. Here, we present the full list of identified Rac1 interactors together with functional annotation of the differentially regulated Rac1 binding partners. In light of this data, we also provide additional insights into known and novel signaling cascades that might account for the GEF-mediated Rac1-driven cellular effects.

## Introduction

Deregulation of Rac1 signaling has been shown to play a major role in tumor initiation, progression and metastasis. ^1-9^ Targeting Rac1 signaling could, therefore, be of potential therapeutic benefit. However, due to the diverse, and sometimes contradictory, functions of Rac1, an effective therapy would require prior determination of how signaling outputs downstream of Rac1 are selected. The pleiotropic effects of Rac1 are mediated mainly through its ability to bind to a multitude of downstream effectors upon activation by Guanine nucleotide Exchange Factors (GEFs).^10,11^ Yet, mechanisms influencing Rac1 downstream signaling specificity, through modulating effector binding, are poorly understood. Interestingly, it has been proposed that GEFs might regulate Rac1 downstream specificity through serving as scaffolding proteins.^11-17^ However, a challenge facing the field was the discrimination between GEF-driven regulation of Rac1 functions and changes induced as a consequence of differences in cell type and/or upstream cues from the Extracellular Matrix (ECM).

To address these limitations we recently performed a study directly comparing the ability of 2 distinct Rac-specific GEFs to regulate Rac1 downstream signaling.^18^ Focusing on Tiam1 and P-Rex1, which have been associated with contrasting migratory phenotypes,^19-24^ we first demonstrated that activation of Rac1 by Tiam1 or P-Rex1, irrespective of cell type or upstream signaling, promotes Rac1 anti- vs. pro-migratory effects, respectively.^18^ Next, to rigorously evaluate the contribution of GEFs in regulating Rac1 signaling through modulating effector binding, we performed a quantitative proteomic analysis of the Rac1 interactome upon activation by either GEF. Via utilizing Stable Isotope Labeling by Amino acids in Cell culture (SILAC) coupled with *StrepII*-FLAG Tandem Affinity Purification (SF-TAP), we showed that activation of Rac1 by either Tiam1 or P-Rex1 results in distinct changes in the Rac1 interactome. Importantly, through characterizing Rac1 binding to protein flightless-1 homolog (FLII), a novel P-Rex1-enriched Rac1 interactor, we demonstrated that, indeed, GEF modulation of the Rac1 interactome is crucial for mediating GEF-specific Rac1 signaling cascades.^18^ We now present the full list of Rac1 interacting proteins identified from the SILAC SF-TAP screens and extend our recently published bioinformatics analysis to include the full list of GEF-specific differentially regulated Rac1 binding partners. We also discuss additional modes by which Tiam1 and P-Rex1 might induce Rac1 anti- versus pro-migratory phenotypes in light of the newly highlighted Rac1 interactors. Thus, together with our recent findings, data presented here provide clear evidence supporting a role of GEFs in regulating Rac1 signaling via modulating Rac1 binding to downstream effectors.^18^ This manuscript also provides a resource for the scientific community entailing a comprehensive dataset of known and novel Rac1 interactors that show altered Rac1 binding in a GEF-dependent manner. Further characterization of these interactions promises to uncover previously unreported GEF-mediated signaling cascades that regulate Rac1-driven cellular functions.

## Results and discussion

### Identification of GEF-mediated Rac1 interactome changes by SILAC SF-TAP proteomic analysis

**Figure 1.**
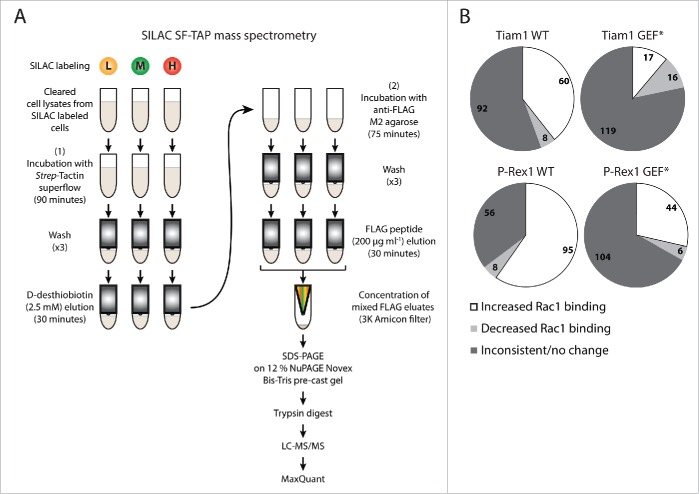
SILAC SF-TAP proteomic analysis. (A) Schematic representation of *StrepII*-FLAG Tandem Affinity Purification (SF-TAP) conducted on NIH3T3 cells transduced with the dual doxycycline (dox)-inducible co-expression system following labeling using Stable Isotope Labeling by Amino acids in Cell culture (SILAC). Three SILAC labeling media containing different lysine (K) and arginine (R) isotope combinations were utilised: L= Light K0+R0; M= Medium K4+R6; H= Heavy K8+R10. Eluates within each set were combined and concentrated. Concentrated mixed eluates were then separated by Sodium Dodecyl Sulfate Polyacrylamide Gel Electrophoresis (SDS-PAGE), digested with trypsin and analyzed by Liquid Chromatography-tandem Mass Spectrometry (LC-MS/MS). Finally, the produced data was processed using the MaxQuant software. (B) Pie chart classifying identified Rac1 binding partners from the SILAC SF-TAP screens into proteins exhibiting inconsistent/unchanged, decreased or increased Rac1 binding upon expression of the indicated GEFs. Numbers reflect proteins displaying indicated Rac1 binding pattern in ≥ 2 SILAC SF-TAP experiments.

As described in our recent publication, to quantitatively evaluate whether Tiam1 and P-Rex1 modulate Rac1-effector binding, NIH3T3 cells transduced with a dual doxycycline (dox)-inducible system for expressing *StrepII*-FLAG-tagged Rac1 (SF-Rac1) alone or together with Wild Type (WT) or GEF-dead mutant (GEF*) versions of Tiam1 or P-Rex1 were labeled using SILAC. Due to the limited number of labeling isotopes we designed 5-way SILAC and reverse SILAC screens in which cells were divided into 2 sets. The first set (set1) included the SF-Rac1 alone expressing cells together with SF-Rac1+Tiam1 WT and SF-Rac1+P-Rex1 WT co-expressing cells, while the second set (set2) included the SF-Rac1 alone expressing cells again, as a common reference, with SF-Rac1+Tiam1 GEF* and SF-Rac1+P-Rex1 GEF* co-expressing cells. This setup allowed the comparison of the proteomes within each set directly as well as between set1 and set2 relative to the common reference.^18^ Following SILAC labeling, lysates from the differentially labeled cells were subjected to SF-TAP^25,26^ to pulldown SF-Rac1 and its binding partners. Eluates within each set were combined and concentrated. Concentrated mixed eluates were then separated by Sodium Dodecyl Sulfate Polyacrylamide Gel Electrophoresis (SDS-PAGE), digested with trypsin and analyzed by Liquid Chromatography-tandem Mass Spectrometry (LC-MS/MS). Finally, the produced data was processed using the MaxQuant software (Fig. 1A).

In total 350 putative Rac1 interactors were identified from 2 SILAC and 2 reverse SILAC SF-TAP screens (Supplementary File 1, Tables S.1-S.3). Importantly, as outlined in our recent manuscript, analysis of the full list of proteins uncovered a number of known and predicted Rac1 binding partners in addition to proteins involved in mediating Rac1 signaling.^18^ This indicated the robustness of the SILAC SF-TAP approach in identifying genuine Rac1 binding partners. We further analyzed a subset of 231 proteins that were associated with SILAC ratios in ≥ 2 experiments (Supplementary File 1, Table S.2). SILAC ratios were then used to determine GEF-induced changes in Rac1 binding by applying a cut-off of ± 1.3-fold-change,^27^ thereby classifying proteins identified under each GEF into interactors that show inconsistent/no change in Rac1 association, proteins with reduced Rac1 binding and proteins with increased Rac1 binding relative to SF-Rac1 alone expressing cells. Only proteins that exhibited consistent GEF-associated Rac1 binding patterns in ≥ 2 experiments were considered for further analysis (Fig. 1B). To identify Rac1 interactors that could account for the distinct GEF-mediated Rac1 downstream effects, we next compared proteins with altered Rac1 binding upon Tiam1 WT or P-Rex1 WT expression to their respective GEF* mutants, thus highlighting proteins that bind mainly to active Rac1. These lists were then cross-referenced to one another to further eliminate overlapping proteins between both GEFs. As described in our recent publication, out of the 231 proteins, 159 showed overlap between the different GEF constructs. Tiam1 WT expression resulted in specific regulation of 15 proteins, Tiam1 GEF* of 14 proteins, P-Rex1 WT of 31 proteins and P-Rex1 GEF* of 12 proteins.^18^ All together, this indicated that Tiam1 and P-Rex1 are indeed capable of specifically modulating the Rac1 interactome.

### Insight into Tiam1-mediated Rac1 anti-migratory effects through modulating Rac1-effector binding

The role of Tiam1-Rac1 signaling in cell migration and invasion is controversial with some reports suggesting an inhibitory role,^8,19,21,28-31^ while others implicating Tiam1 in promoting migration.^32-40^ This is mainly due to the fact that Tiam1-mediated activation of Rac1 regulates both actin cytoskeleton rearrangements as well as cell-cell and cell-matrix adhesions. It has thus been proposed that the effects of Tiam1-Rac1 signaling are cell type and substrate specific.^41,42^ A clear example of such influence is evident from the ability of Tiam1, through Rac1 activation, to enhance E-cadherin-mediated cell-cell contacts when Ras transformed MDCKII (MDCK-f3) cells are plated on fibronectin or laminin, while promoting migration through inducing membrane ruffling and lamellipodia formation when cells are plated on different types of collagen.^42^ However, via bypassing ECM signaling cues, we have recently shown that expression of Tiam1 in different cell lines results exclusively in Rac1-driven anti-migratory phenotypes.^18^ This suggests that Tiam1, in the absence of ECM signaling interference, might function predominantly to inhibit Rac1-driven cell migration and invasion.

**Figure 2.**
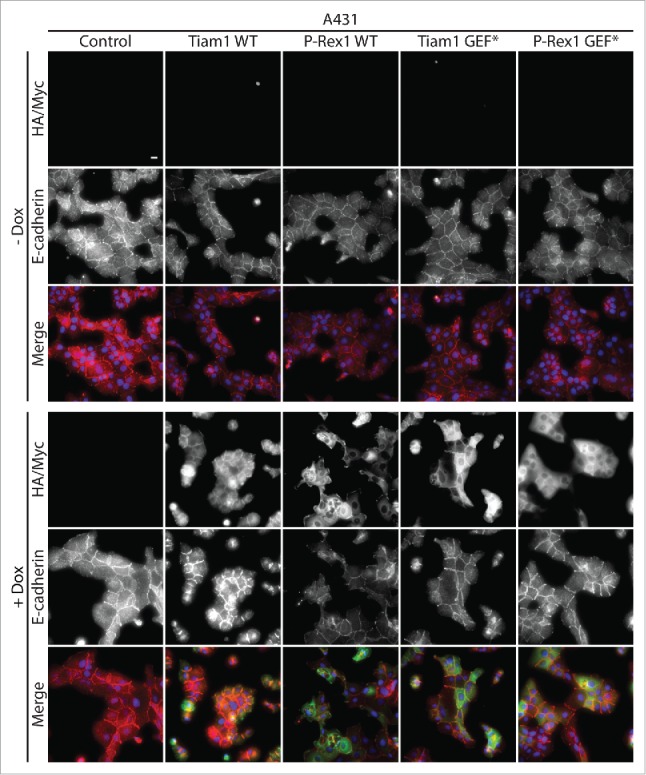
Activation of Rac1 by Tiam1 but not P-Rex1 increases E-cadherin levels at cell-cell contacts in A431 cells. A431 cells transduced with the doxycycline (dox)-inducible GEF expression system were treated with ethanol (- dox) or 1 μg ml ^−1^ dox (+ dox) for 24 hours. Cells were then fixed and localization of E-cadherin was detected by immunofluorescence. Fluorescence markers against HA-tagged Tiam1 WT/GEF* or Myc-tagged P-Rex1 WT/GEF* were used to detect the expression of the respective GEF constructs upon dox induction. DAPI was used to visualize the nuclei. Scale bar = 20 µM.

Tiam1-Rac1 anti-migratory effects are largely linked to their role in promoting cadherin-mediated cell-cell contacts. ^19,21,29,30^ We recently demonstrated that expression of Tiam1 WT but not GEF* mutant results in increased cellular aggregation and enhanced actin localization at cell-cell contacts to induce a compact epithelial-like morphology in A431 cells.^18^ We now show that Tiam1 WT expression, but not P-Rex1 WT or the GEF* mutants, enhances E-cadherin recruitment to cell-cell contacts (Fig. 2). Thus, consistent with previous reports, our data strongly suggests that Tiam1-mediated Rac1 anti-migratory effects might be a consequence of stronger cell-cell contacts.

**Figure 3.**
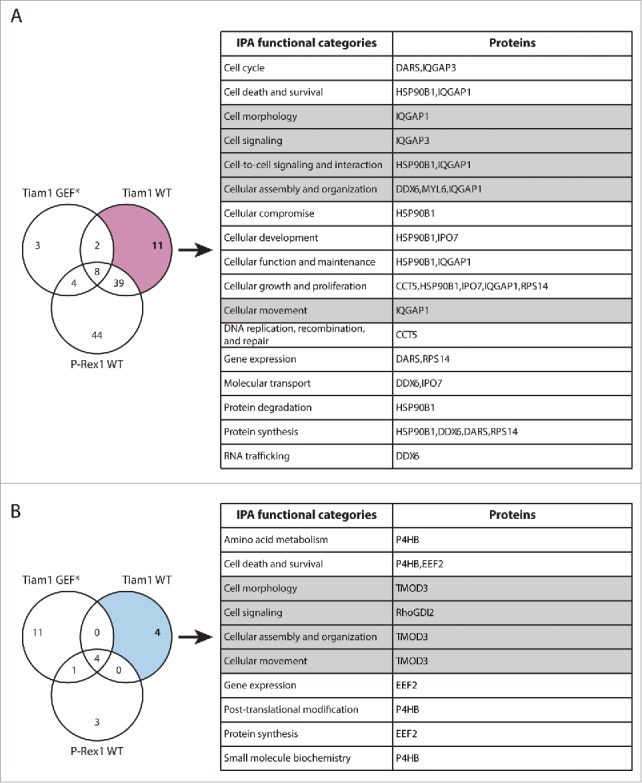
Functional classification of proteins with Tiam1 WT-specific changes in Rac1 binding. (A) Venn diagram comparing proteins with increased Rac1 binding in ≥ 2 SILAC SF-TAP experiments upon expression of indicated GEF constructs. Tiam1 Wild Type (WT)-specific proteins are outlined. (B) Venn diagram comparing proteins with decreased Rac1 binding in ≥ 2 SILAC SF-TAP experiments upon expression of indicated GEF constructs. Tiam1 WT-specific proteins are outlined. For A and B the associated tables show clustering of the Tiam1 WT-specific proteins with increased or decreased Rac1 binding, respectively, according to their cellular functions based on Ingenuity Integrated Pathway Analysis (IPA). Full protein names and SILAC ratios are outlined in Supplementary File 1. Proteins in highlighted categories were presented as part of a heat map in our recent publication. ^18^

**Figure 4.**
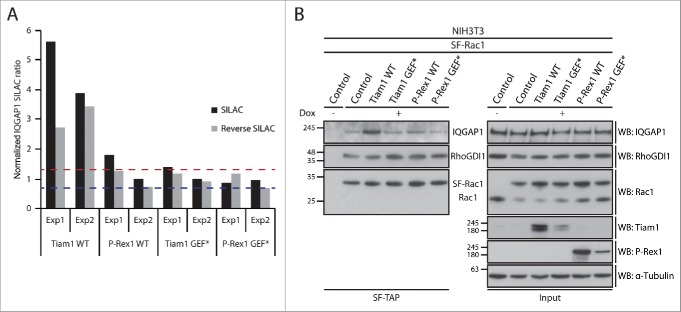
Rac1-IQGAP1 interaction is enhanced upon activation of Rac1 by Tiam1. (A) Graph representing normalized IQGAP1 SILAC ratios relative to NIH3T3 cells expressing SF-Rac1 alone from 2 SILAC and 2 reverse SILAC SF-TAP screens (Exp1 and Exp2). Dashed lines represent the upper (red line) and lower (blue line) bounds of the cut-off applied to infer changes in Rac1 binding. (B) *StrepII*-FLAG Tandem Affinity Purification (SF-TAP) of SF-Rac1 from NIH3T3 cells treated with ethanol (- dox) or 1 μg ml^−1^ doxycycline (+ dox) to induce expression of SF Rac1 alone (control) or together with the indicated GEFs. Co-precipitated endogenous IQGAP1 and RhoGDI1 were detected by protein gel blot analysis. α-Tubulin was used as a loading control.

To link these observations to Rac1 interactors that exhibited Tiam1 WT-specific changes in Rac1 binding, we utilised Ingenuity Integrated Pathway Analysis (IPA) to categorise these proteins according to their cellular functions (Fig. 3). Among the identified proteins, the known Rac1 effector, IQGAP1, was associated with a number of functional categories that could account for Tiam1 WT-mediated Rac1-driven effects (Fig. 3A). SILAC ratios indicated that IQGAP1 exhibits increased Rac1 binding specifically upon expression of Tiam1 WT (Fig. 4A and Supplementary File 1, highlighted in Table S.2). To further validate the screen we performed SF-TAP of SF-Rac1 from NIH3T3 cells transduced with the dual dox-inducible co-expression system. Analysis of IQGAP1 levels co-precipitated with SF-Rac1 revealed that, as indicated by the IQGAP1 associated SILAC ratios, expression of Tiam1 WT, but not P-Rex1 WT or the GEF* mutants, results in increased Rac1-IQGAP1 binding. In contrast, consistent with our recent observations,^18^ co-expression of either GEF with SF-Rac1 did not induce any changes in Rac1-RhoGDI1 binding (Fig. 4B).

IQGAP1 is implicated in various signaling processes, such as cytoskeleton reorganization, cell-cell adhesion and proliferation.^43^ It has also been shown that, through its ability to bind to actin, IQGAP1 links Rac1 and Cdc42 to the actin cytoskeleton.^44,45^ Moreover, IQGAP1 can regulate actin assembly via serving as a calmodulin-regulated barbed-end actin capping protein.^46^ Given the role of IQGAP1 in regulating the actin cytoskeleton, increased Rac1 binding following activation by Tiam1 could be important in mediating the extensive membrane ruffling and increased actin accumulation at cell-cell contacts associated with Tiam1 WT expression.^18^

Intriguingly, IQGAP1 has been shown to promote migration through disrupting the interaction between E-cadherin and its binding partner β-catenin, which in turn mediates the disassembly of cell-cell contacts.^47,48^ However, given our recent findings indicating that Tiam1 WT expression results in increased cellular aggregation and membrane ruffling accompanied by reduced cell migration, we recently proposed that the increased Rac1-IQGAP1 interaction mediated by Tiam1 might negatively regulate IQGAP1 function.^18^ Indeed, increased activation of Rac1 and binding to IQGAP1 has been shown to inhibit IQGAP1-β-catenin interaction, as well as impede IQGAP1-mediated α-catenin translocation from the cadherin-catenin complex.^48-50^ Alternatively, it is possible that under the cellular context investigated IQGAP1 is important for maintaining cell-cell contacts. Consistent with this, IQGAP1 has been implicated in establishing Vascular Endothelial (VE)-cadherin-mediated cell-cell contacts in human endothelial cells^51^ and enhancing cell-cell contacts in endocrine cells.^52^ In either case, it seems that through stimulating Rac1-IQGAP1 binding above normal levels, Tiam1 directs IQGAP1 to enhance cell-cell contacts, thereby reducing cell migration.

In addition to its function as a regulator of cell-cell contacts, IQGAP1 has been shown to interact with components of the exocyst complex, including Sec3, Sec8 and Exo70 and is thus believed to play an important role in exocytosis.^53,54^ Interestingly, Tiam1-mediated Rac1 activation has been linked to increased transcriptional and post-transcriptional upregulation of Tissue Inhibitor of Metalloproteinase-1 (TIMP-1) and TIMP-2, respectively, while not affecting the secreted levels or activity of Matrix Metalloproteinase-9 (MMP-9) or MMP-2.^28^ It is, therefore, possible that an increased interaction between Rac1 and IQGAP1 plays a role in promoting the secretion of TIMP-1 and TIMP-2 by exocytosis to protect against ECM degradation, thereby supressing cell invasion. Thus it would be interesting to analyze the extracellular levels of TIMPs upon Tiam1 expression and assess its dependency on IQGAP1. Understanding this mechanism could have important implications on Rac1-driven cancer dissemination.

### Insight into P-Rex1-mediated Rac1 pro-migratory effects through modulating Rac1-effector binding

**Figure 5.**
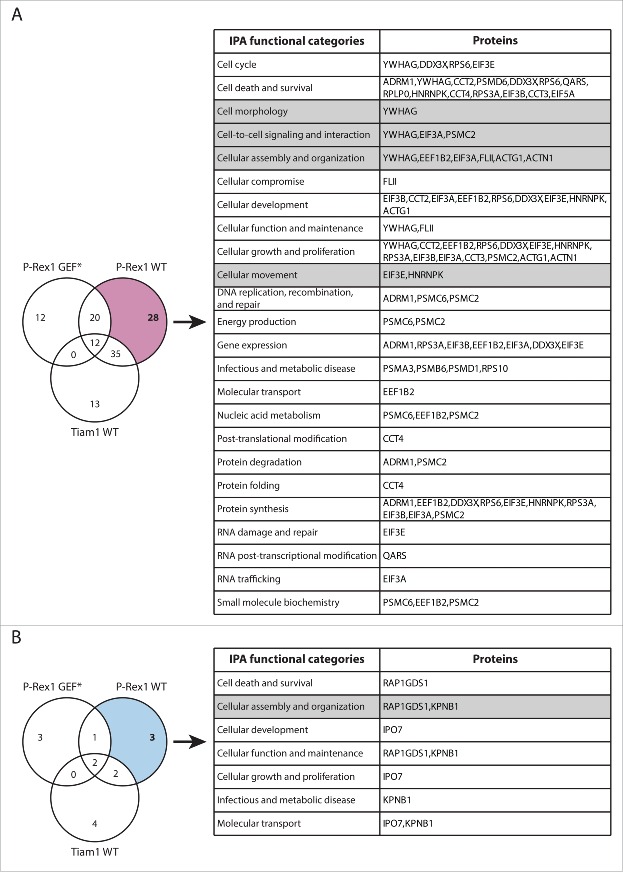
Functional classification of proteins with P-Rex1 WT-specific changes in Rac1 binding. (A) Venn diagram comparing proteins with increased Rac1 binding in ≥ 2 SILAC SF-TAP experiments upon expression of indicated GEF constructs. P-Rex1 Wild Type (WT)-specific proteins are outlined. (B) Venn diagram comparing proteins with decreased Rac1 binding in ≥ 2 SILAC SF-TAP experiments upon expression of indicated GEF constructs. P-Rex1 WT-specific proteins are outlined. For A and B the associated tables show clustering of the P-Rex1 WT-specific proteins with increased or decreased Rac1 binding, respectively, according to their cellular functions based on Ingenuity Integrated Pathway Analysis (IPA). Full protein names and SILAC ratios are outlined in Supplementary File 1. Proteins in highlighted categories were presented as part of a heat map in our recent publication.^18^

Functional analysis of the identified Rac1 interactors also highlighted a number of signaling proteins involved in promoting cell migration. Proteins displaying P-Rex1 WT-specific altered Rac1 binding are of particular interest as further characterization of their role in Rac1 signaling promises to shed light on novel regulatory mechanisms by which Rac1 promotes migration and cancer dissemination (Fig. 5). Among the P-Rex1 WT-specific enriched Rac1 binding partners (Fig. 5A), we recently identified the gelsolin superfamily member, FLII as a novel Rac1 interactor. Functional characterization of this interaction revealed that P-Rex1 promotes Rac1-FLII binding, which facilitates P-Rex1-FLII-dependent and RhoA-ROCK-independent cell contraction required for mediating Rac1-driven cell migration. Thus, this study provided clear evidence supporting the role of P-Rex1 in modulating Rac1 downstream function through dictating specific Rac1-effector protein complexes.^18^

In addition to FLII, SILAC ratios also indicated increased Rac1 binding to α-actinin 1 (Actn1) upon P-Rex1 WT expression (Fig. 5A and Supplementary File 1, highlighted in Table S.2). Interestingly, both Actn1 and Rac1 have been shown to contribute to the formation of non-contractile dorsal stress fibers that promote cell migration and spreading.^55^ This hints at another potential mechanism by which P-Rex1 might promote Rac1 pro-migratory phenotypes through enhancing Rac1-Actn1 binding to stimulate dorsal stress fiber formation.

The SILAC SF-TAP screens also shed light on more anticipated mechanisms by which P-Rex1 might promote Rac1 pro-migratory effects. Rac1 has been shown to contribute to lamellipodia formation through activating the Arp2/3 protein complex.^56^ Given that P-Rex1 WT expressing cells are associated with the formation of actin-rich thin extended membrane protrusions,^18^ it is possible that P-Rex1 utilizes the Arp2/3 protein complex machinery to promote Rac1-driven actin polymerization necessary for the formation of these protrusions. Consistent with this notion, 3 subunits of the Arp2/3 protein complex, namely Arpc1b, Arpc2 and Arp3, were identified as increased Rac1 binding partners under P-Rex1 WT but not P-Rex1 GEF* in a single SILAC SF-TAP experiment. More interestingly, these components show decreased Rac1 binding upon expression of Tiam1 WT as inferred from their SILAC ratios (Supplementary File 1, highlighted in Table S.1). It is, therefore, likely that the ability of Tiam1 and P-Rex1 to differentially modulate Rac1 binding to the Arp2/3 protein complex is important for the observed differential effects upon activation of Rac1 by either GEF.

### GEF activity-independent scaffolding role of GEFs

Through deciphering the role of P-Rex1-mediated Rac1-FLII interaction in promoting cell migration we uncovered a scaffolding function of P-Rex1. Biochemical analysis indicated that P-Rex1 WT stimulates the Rac1-FLII interaction through directly binding to FLII. Importantly, both P-Rex1 WT and its GEF* mutant exhibited comparable FLII interaction, suggesting that while Rac1 activation is important for Rac1-FLII binding, the P-Rex1 GEF activity is not required for the P-Rex1-FLII interaction. This provided strong evidence suggesting a GEF activity-independent scaffolding role of GEFs in cells.^18^

**Figure 6.**
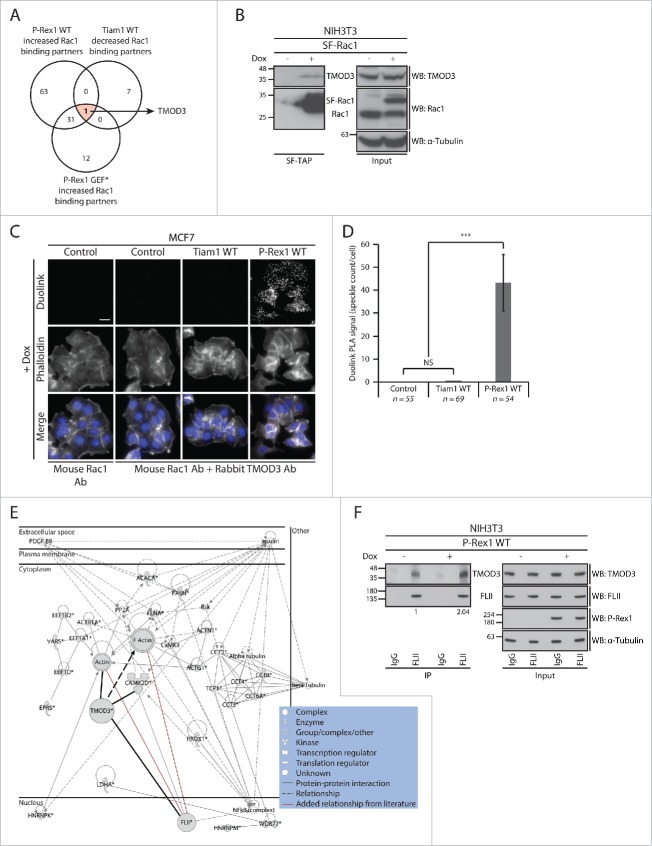
TMOD3 is a novel P-Rex1-enriched Rac1 interactor that binds to FLII in a P-Rex1-dependent manner. (A) Venn diagram comparing proteins that show increased Rac1 binding under P-Rex1 Wild Type (WT) and P-Rex1 GEF-dead mutant (GEF*) together with decreased Rac1 binding under Tiam1 WT expression in ≥ 2 SILAC SF-TAP experiments. TMOD3 is the only protein that exhibits opposing Rac1 binding patterns upon P-Rex1 WT/GEF* and Tiam1 WT expression. (B) *StrepII*-FLAG Tandem Affinity Purification (SF-TAP) of SF-Rac1 from NIH3T3 cells treated with ethanol (- dox) or 1 μg ml ^−1^ doxycycline (+ dox) to induce expression of SF-Rac1. Co-precipitated endogenous TMOD3 was detected by protein gel analysis. α-Tubulin was used as a loading control. (C) Representative immunofluorescence images of MCF7 cells subjected to the Duolink *in situ* Proximity Ligation Assay (PLA) following treatment with 1 µg ml ^−1^ dox (+ dox) for 24 hours to induce expression of indicated GEF constructs. Phalloidin and DAPI were used to visualize the actin cytoskeleton and nuclei, respectively. Scale bar= 20 µm. (D) Quantification of the average Duolink PLA signal from indicated number of MCF7 cells described in C ± s.e.m. Student's t-test was performed to determine statistical significance and p-values are shown on graph. NS= non-significant; ***= p ≤ 0.001. (E) Ingenuity protein-protein network cluster of proteins showing increased Rac1 binding upon expression of P-Rex1 WT in ≥ 2 SILAC SF-TAP experiments. Network shown represents one of the generated networks and displays relationships between proteins identified in the screen (*) with other proteins from the Ingenuity database that have similar functions. Additional relationships (red lines) were incorporated in the network based on information from the literature. Bold lines were used to highlight TMOD3 protein interactions and relationships. (F) Endogenous FLII immunoprecipitation (IP) from NIH3T3 cells transduced with the dox-inducible P-Rex1 WT expression system following treatment with ethanol (- dox) or 1 µg ml ^−1^ dox (+ dox). Co-precipitated endogenous TMOD3 was detected by protein gel blot analysis. α-Tubulin was used as a loading control. Quantification of FLII-bound TMOD3 was assessed using the ImageJ software and normalized to α-Tubulin and total TMOD3 levels in the input. The normalized integrated density detected in the – dox FLII IP sample was set as one highlighting a 2-fold increase in FLII-TMOD3 binding upon expression of P-Rex1 WT as indicated.

To gain additional insight into the P-Rex1 GEF scaffolding function, we screened the list of Rac1 interactors for proteins with opposing Rac1 binding patterns upon expression of Tiam1 and P-Rex1, irrespective of their GEF activity. Among the identified proteins, the actin capping protein tropomodulin-3 (TMOD3), was the only protein associated with increased SILAC ratios upon P-Rex1 expression and decreased SILAC ratios upon Tiam1 WT expression (Fig. 6A and Supplementary File 1, highlighted in Table S.2). Using protein gel blot analysis we demonstrated that SF-Rac1 co-precipitates endogenous TMOD3 in NIH3T3 cells expressing SF-Rac1 (Fig. 6B). Additionally, we also confirmed that expression of P-Rex1 WT in MCF7 cells, unlike Tiam1 WT, is associated with an increased Rac1-TMOD3 interaction as indicated by the Duolink *in situ* Proximity Ligation Assay (PLA) used to visualize the endogenous interaction between Rac1 and TMOD3 (Fig. 6C, D). Taken together, this indicates that TMOD3 is a *bona fide* Rac1 interactor that exhibits enhanced Rac1 binding in a P-Rex1-dependent manner.

Intriguingly, according to the SILAC SF-TAP screens, expression of both P-Rex1 WT and P-Rex1 GEF* was associated with increased Rac1-TMOD3 binding (Fig. 6A and Supplementary File 1, highlighted in Table S.2). This suggests that activation of Rac1 is not important for this interaction to occur, and implies that TMOD3 may not directly contribute to the observed P-Rex1-Rac1-driven cellular phenotypes. Nevertheless, analysis of Ingenuity IPA generated protein-protein networks indicated a potential interaction between TMOD3 and FLII (Fig. 6E).^57^ Indeed, further biochemical analysis using NIH3T3 cells expressing P-Rex1 WT in a dox-inducible manner revealed that TMOD3 and FLII interact on an endogenous level and that expression of P-Rex1 WT stimulates this interaction (Fig. 6F). This hints at a potential role of GEFs as scaffolding proteins not only for Rac1 but also for Rac1 effectors. The increased FLII-TMOD3 interaction, may thus play a Rac1-independent role that is yet to be elucidated, or it might be important for the formation of the recently described P-Rex1-FLII-Rac1 complex, thereby mediating specific P-Rex1-Rac1-driven cellular effects upon Rac1 activation.^18^ TMOD3 might, therefore, function as a GEF-regulated scaffolding protein that helps bring other proteins in close proximity to Rac1, thus priming them for binding once Rac1 is in the active form.

**Figure 7.**
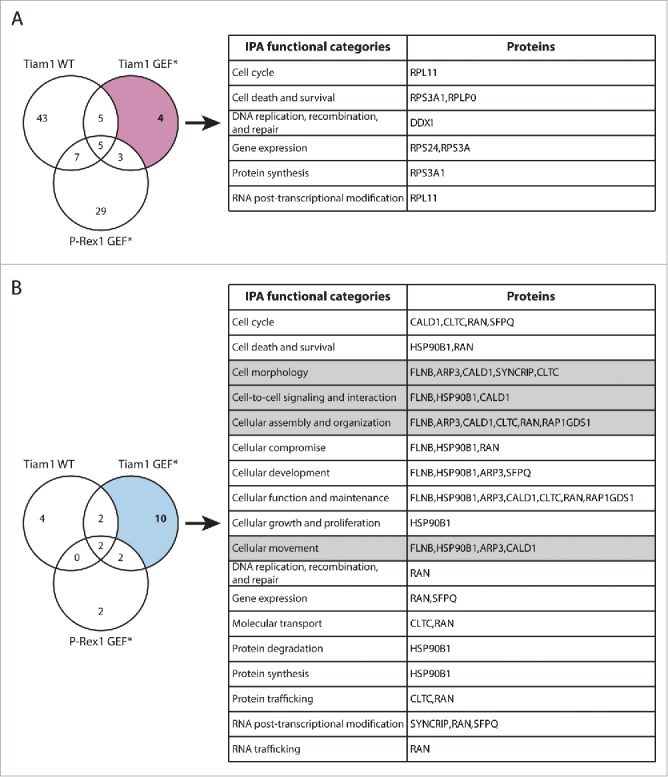
Functional classification of proteins with Tiam1 GEF*-specific changes in Rac1 binding. (A) Venn diagram comparing proteins with increased Rac1 binding in ≥ 2 SILAC SF-TAP experiments upon expression of indicated GEF constructs. Tiam1 GEF-dead mutant (GEF*)-specific proteins are outlined. (B) Venn diagram comparing proteins with decreased Rac1 binding in ≥ 2 SILAC SF-TAP experiments upon expression of indicated GEF constructs. Tiam1 GEF*-specific proteins are outlined. For A and B the associated tables show clustering of the Tiam1 GEF*-specific proteins with increased or decreased Rac1 binding, respectively, according to their cellular functions based on Ingenuity Integrated Pathway Analysis (IPA). Full protein names and SILAC ratios are outlined in Supplementary File 1. Proteins in highlighted categories were presented as part of a heat map in our recent publication. ^18^

**Figure 8.**
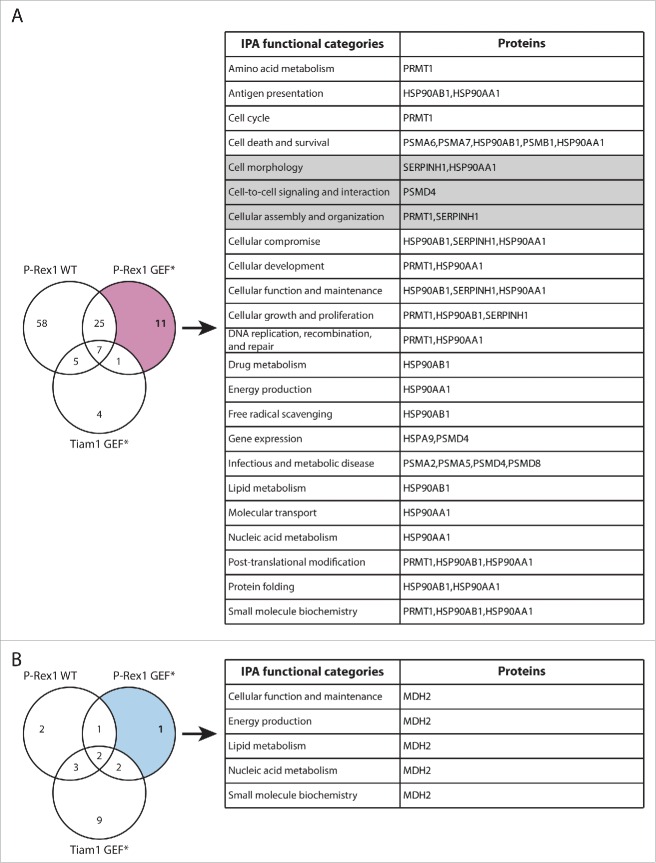
Functional classification of proteins with P-Rex1 GEF*-specific changes in Rac1 binding. (A) Venn diagram comparing proteins with increased Rac1 binding in ≥ 2 SILAC SF-TAP experiments upon expression of indicated GEF constructs. P-Rex1 GEF-dead mutant (GEF*)-specific proteins are outlined. (B) Venn diagram comparing proteins with decreased Rac1 binding in ≥ 2 SILAC SF-TAP experiments upon expression of indicated GEF constructs. P-Rex1 GEF*-specific proteins are outlined. For A and B the associated tables show clustering of the P-Rex1 GEF*-specific proteins with increased or decreased Rac1 binding, respectively, according to their cellular functions based on Ingenuity Integrated Pathway Analysis (IPA). Full protein names and SILAC ratios are outlined in Supplementary File 1. Proteins in highlighted categories were presented as part of a heat map in our recent publication.^18^

In addition to TMOD3, the SILAC SF-TAP screens also highlighted a number of other proteins that exhibited GEF-specific changes in Rac1 binding upon expression of Tiam1 GEF* and P-Rex1 GEF*, further supporting a role of GEFs as scaffolding proteins, irrespective of Rac1 activation. Functional analysis of these proteins using Ingenuity IPA analysis suggests that, in addition to modulating Rac1-effector binding, GEFs might also mediate Rac1 interaction with regulatory proteins potentially influencing Rac1 levels, subcellular localization and post-translational modification (Fig. 7 and Fig. 8). Therefore, analysis of these proteins might also shed light on additional modes by which GEFs modulate Rac1 signaling through spatial and temporal regulation.

## Conclusions

Due to the complexity of Rac1 signaling under normal and pathological conditions, it is crucial to identify factors that contribute to its downstream specificity. Through conducting a comparative analysis of Rac1-driven cellular functions upon activation by 2 Rac-specific GEFs, Tiam1 and P-Rex1, we provide clear evidence highlighting their role in dictating differential Rac1-dependent cellular processes. Importantly, we link these differential effects to the ability of each GEF to induce specific changes to the Rac1 interactome, through serving as scaffolding proteins. Additionally, our data suggest that this scaffolding function might extend, not only to Rac1 but also to Rac1 binding partners. This expands the biological significance of GEFs beyond their ability to activate Rac1. GEFs might also contribute to Rac1 spatial and temporal regulation, irrespective of Rac1 activation status. Therefore, functional analysis provided in this manuscript serves as a mining tool for known and novel Rac1 interactors that are regulated through GEFs. Future studies of these proteins promise to provide novel mechanistic insight into GEF-mediated Rac1 signaling cascades that will aid in further dissecting the role of GEFs in dictating Rac1 signaling.

## Author contributions

HM designed and performed the majority of the experiments and Ingenuity IPA analysis and wrote the manuscript. AC performed the mass spectrometry analysis on samples submitted following SILAC labeling and SF-TAP. BM provided the expertise and equipment required for the mass spectrometry analysis. AM devised and supervised the project and edited the manuscript.

## Competing financial interests

The authors declare no competing financial interests.

## Methods

### Antibodies

Details of antibodies used in this study are summarised below.

**Table:** 

Antibody	Species	Manufacturer	Dilution/Remarks
Alexa Fluor 488	N/A	Thermo Fisher Scientific, A11006	1:500 for immunofluorescence
Alexa Fluor 568 Phalloidin	N/A	Thermo Fisher Scientific, A12380	1:100 for immunofluorescence
Alexa Fluor 647	N/A	Thermo Fisher Scientific, A31573	1:500 for immunofluorescence
Anti-HA	Rabbit	Abcam, ab13834	1:500 for immunofluorescence
Anti-Mouse-HRP	N/A	GE Healthcare, RPN4201	1:5000 for protein gel blot
Anti-Rabbit-HRP	N/A	GE Healthcare, RPN4301	1:5000 for protein gel blot
E-cadherin, DECMA-1	Rat	Abcam, ab11512	1:100 for immunofluorescence
Flightless I (116.40)	Mouse	Santa Cruz, sc-21716	1:1000 for western blot 2.5-12.5 μg for immunoprecipitation
FLII	Rabbit	Sigma-Aldrich, HPA007084	1:1000 for protein gel blot
IQGAP1 (24)	Mouse	BD Biosciences, 610611	1:1000 for protein gel blot
Myc-488 conjugated	N/A	Merck Millipore, 16-224	1:100 for immunofluorescence
P-Rex1	Rabbit	Sigma-Aldrich, HPA001927	1:1000 for protein gel blot
Rac1 (102)	Mouse	BD Biosciences, 610650	1:1000 for protein gel blot
RhoGDI1 (FL-204)	Rabbit	Santa Cruz, sc-33201	1:1000 for protein gel blot
Tiam1	Rabbit	Bethyl, A300-099A	1:1000 for protein gel blot
TMOD3	Rabbit	Sigma-Aldrich, HPA001849	1:1000 for western blot 1:100 for immunofluorescence
α-Tubulin (DM1A)	Mouse	Sigma-Aldrich, T9026	1:5000 for protein gel blot

### Constructs and cell lines

Please refer to Marei et al.^18^ for details of expression plasmids and cell lines utilised in this study.

### Immunofluorescence

A431 cells grown on coverslips in the presence of ethanol (- dox) or 1 µg ml ^−1^ doxycycline (+ dox) for 24 hours were fixed, mounted and imaged using the Low Light microscope system as described by Marei et al.^18^ Rat anti-E-cadherin (DECMA-1; Abcam, ab11512) was used to visualize GEF-induced changes to E-cadherin localization. Fluorescence markers against HA-tagged Tiam1 WT/GEF* or Myc-tagged P-Rex1 WT/GEF* were used to detect the expression of the respective GEFs upon dox induction. DAPI was used to stain the nuclei.

### Duolink *in situ* Proximity Ligation Assay (PLA)

MCF7 cells transduced with the GEF dox-inducible system were seeded on glass coverslips in the presence of 1 µg ml ^−1^ dox for 24 hours. Cells were then fixed in 4 % formaldehyde and subjected to the Duolink *in situ* Proximity Ligation Assay (PLA). Mouse anti-Rac1 (BD Biosciences, 610650) and rabbit anti-TMOD3 (Sigma-Aldrich, HPA001849) were used together with the respective Duolink *in situ* PLA probes (Olink Bioscience, anti-mouse 92004-0100, anti-rabbit 92002-0100) and the Duolink *in situ* detection reagent kit (Olink Bioscience, 92013-0100, 92014-0100) according to manufacturer's instructions. Coverslips were mounted onto slides using ProLong Gold antifade reagent with DAPI stain (Life Technologies, P36935). Phalloidin and DAPI were used to visualize the actin cytoskeleton and the nuclei, respectively. Images were captured on the Low Light microscope system using fixed focus and exposure settings to ensure that differences detected in the Duolink signal are only due to changes in Rac1-TMOD3 binding. Quantification of the Duolink signal was performed as described by Marei et al.^18^

### Immunoprecipitation and *StrepII*-FLAG Tandem Affinity Purification (SF-TAP)

Cells were lysed in lysis buffer [30 mM Tris pH 7.4, 150 mM NaCl, 0.5 % Nonidet P40 (v/v), 1 % protease inhibitor cocktail (v/v), 1 % phosphatase inhibitor cocktails 1 and 2 (v/v) in dH_2_O]. For immunoprecipitation, lysates were incubated with 20-50 μl antibody pre-bound to GammaBind G Sepharose beads (GE Healthcare, 17-0885-01) for 2 hours at 4°C, washed and resuspended in 25 μl 2x SDS-PAGE sample buffer [50 % NuPAGE LDS sample buffer 4X (Life Technologies, NP0008) (v/v), 20 % NuPAGE sample reducing agent 10X (Life Technologies, NP0004) (v/v) in dH_2_O] for protein gel analysis. The SF-TAP was performed as previously described by Gloeckner et al.^25^ with minor modifications as outlined in Fig. 1A and in our recent manuscript.^18^

### Protein gel analysis

Samples were resolved using NuPAGE Novex Bis-Tris pre-cast gels (Life Technologies, 12 % 10-well NP0341BOX) using the BLUeye Pre-Stained Protein Ladder (Geneflow Ltd, S6-0024) as a protein size reference. Proteins were transferred onto Immobolin PVDF membranes (Millipore, IPFL00010) and protein gel blot analysis was performed using indicated antibodies and visualized on Hyperfilm ECL (GE Healthcare, 28-9068) using the ECL western blotting analysis system (GE Healthcare, RPN2109). Adobe Photoshop software was used to crop full blots and band integrated densities were quantified using the ImageJ software.

### SILAC labeling and mass spectrometry protein identification

NIH3T3 cells transduced with the dual dox-inducible co-expression system were labeled and processed as described by Marei et al.^18^

### SILAC SF-TAP screen validation, ratio classification and Ingenuity analysis

Protein lists generated from the SILAC SF-TAP experiments were analyzed using available protein databases, such as the Human Protein Reference Database (HPRD),^58^ NetPath^59^ and the Human Protein-Protein Interaction Predictions (PIPs) database.^60,61^ These databases coupled with an extensive literature search were used to determine the efficiency of the SILAC SF-TAP screens in identifying *bona fide* Rac1 binding partners, through classifying identified proteins into known Rac1 binding partners, predicted Rac1 binding partners and proteins involved in Rac1 signaling as outlined in our recent manuscript.^18^ Proteins with SILAC ratios in ≥ 2 experiments were classified using a cut-off of ± 1.3 SILAC ratio fold-change ^27^ to identify proteins that show changes in Rac1 binding compared to control cells expressing SF-Rac1 alone. Generated protein lists for each GEF were analyzed using the Ingenuity IPA tool (Ingenuity Systems, http://www.ingenuity.com). This allowed the clustering of proteins according to their functional roles in cells. Ingenuity protein-protein networks were generated for protein lists displaying altered Rac1 binding identified under each GEF.

## Supplementary Material

1183852_Supplemental_Material.zip

## Abbreviations

Actn1: Alpha-actinin 1
Arp2/3: Actin-related protein 2/3
Arp3: Actin-related protein 3
Arpc1b: Actin-related protein 2/3 complex, subunit 1B, 41 kDa
Arpc2: Actin-related protein 2/3 complex, subunit 2, 34 kDa
CHL1: Homo sapiens skin melanoma cell line
Dox: Doxycycline
ECM: Extracellular Matrix
FLII: Protein flightless-1 homolog
GEF: Guanine nucleotide Exchange Factor
GEF*: GEF-dead mutant
GTPase: Guanosine Triphosphate Phosphohydrolase
H: Heavy SILAC media
HPRD: Human Protein Reference Database
IQGAP1: Ras GTPase-activating-like protein 1
K: Lysine
KDa: Kilodaltons
L: Light SILAC media
LC-MS/MS: Liquid Chromatography-tandem Mass Spectrometry
M: Medium SILAC media
MCF7: Michigan Cancer Foundation-7 breast cancer cell line
MDCK-f3: Fibroblastoid MDCKII cells
MDCKII: Madin-Darby Canine Kidney II
MMP: Matrix Metalloproteinase
NIH3T3: National Institutes of Health 3-day Transfer, Inoculum 3 × 10^5^ cells
P-Rex1: Phosphatidylinositol-3,4,5-trisphosphate-dependent Rac exchange factor 1
PIPs: Human Protein-Protein Interaction Predictions (PIPs) database
PLA: Proximity Ligation Assay
R: Arginine
Rac1: Ras-related C3 botulinum toxin substrate 1
Rho A: Ras homolog gene family member A
RhoGDI1: Rho GDP-dissociation inhibitor 1
ROCK: Rho-associated serine/threonie kinase
SDS- PAGE: Sodium Dodecyl Sulfate Polyacrylamide Gel Electrophoresis
SF: *StrepII*-FLAG
SILAC: Stable Isotope Labeling by Amino acids in Cell culture
TAP: Tandem Affinity Purification
Tiam1: T-cell lymphoma invasion and metastasis-1
TIMP: Tissue Inhibitor of Metalloproteinase
TMOD3: Tropomodulin-3
VE: Vascular Endothelial
WT: Wild Type
