# Supplementary material for: Proteomic analysis of Rac1 signaling regulation by guanine nucleotide exchange factors
Source: Cell Cycle. 2016 May 6;15(15):1961–74. doi: 10.1080/15384101.2016.1183852 (PMC4968972; doi:10.1080/15384101.2016.1183852)
Supplement: 1183852_Supplemental_Material.zip [file kccy-15-15-1183852-s001.zip › 1183852_Supplemental Material.docx]

Table S.1 highlights the full list of proteins identified from the SILAC SF-TAP screens with the associated SILAC ratios

Table S.2 highlights the list of proteins with SILAC ratios in two or more SILAC SF-TAP screens

Table S.3 provides the raw MaxQuant file generated from the SILAC SF-TAP screens
